# Thirteenth International Medical Congress, Paris, August 2-9, 1900

**Published:** 1899-12

**Authors:** Henry Barton Jacobs

**Affiliations:** Secretary American National Com.; No. 3 W. Franklin St., Baltimore, Md.


### Society Notes.

### Thirteenth International Medical Congress, Paris, August 2-9, 1900.

AMERICAN NATIONAL COMMITTEE,
DR. WILLIAM OSLER, Chairman.
DR. HENRY BARTON JACOBS, Secretary.

BALTIMORE, MD., Nov. 1, 1899.

DEAR DOCTOR: The American National Committee of the Thirteenth International Medical Congress, to be held in Paris from August 2 to 9, 1900, in connection with the French Exposition, has been organized as above indicated.

All Doctors of Medicine are entitled to membership in this Congress by making the proper application and paying the sum of $5.00. The Secretary-General in Paris has instructed the American National Committee to receive the applications of American physicians, and for this purpose a blank form is enclosed, upon which is to be written full name and address, degrees and any position of note held, together with the Section of the Congress to which the writer wishes to belong. A visiting card should also be appended. These forms, with the $5.00, are to be returned to the Secretary of the National Committee. He in turn will send receipt and forward the slips and money to Paris, where they will be registered, and in due course of time a card of admission to the Congress mailed to each applicant.

The Committee hopes the American representation in this extremely important Medical Congress may be as large as possible, and they would urge every member of the profession to enter his name for membership, this alone entitling him to receive a digest of the full proceedings of the Congress and the printed report* of the section to which he belongs.

---

*Communications respecting the delivery of these reports to members to be addressed to M. Masson, publisher of the proceedings of the Congress, 120, boulevard St. Germain, Paris.

Members desiring to present papers will forward the title and a résumé, before May 1, 1900, to the Secretary of the section to which they belong, for each Sectional Committee reserves to itself the right of drawing up its own working program. Papers are limited to fifteen minutes.

Very sincerely yours,

HENRY BARTON JACOBS,

Secretary American National Com.

No. 3 W. Franklin St., Baltimore, Md.

[Sections, etc., omitted for lack of space. Physicians interested can procure same from the secretary.—ED.]

